# Can Older Adult Patients with Hip Fractures Have Their Discharge Destination Predicted by Physical Performance Measures?

**DOI:** 10.3390/medicina60071017

**Published:** 2024-06-21

**Authors:** Min-A Seok, Jun-Hwan Choi, Young-Ho Roh, So-Young Lee, Hyun-Jung Lee

**Affiliations:** 1 Department of Rehabilitation Medicine, Regional Rheumatoid and Degenerative Arthritis Center, Jeju National University Hospital, Jeju National University College of Medicine, Jeju 63241, Republic of Korea; mmina5137@gmail.com (M.-A.S.); bluelsy900@hanmail.net (S.-Y.L.); sigano@hanmail.net (H.-J.L.); 2 Department of Orthopedic Surgery, Regional Rheumatoid and Degenerative Arthritis Center, Jeju National University Hospital, Jeju National University College of Medicine, Jeju 63241, Republic of Korea; shdudgh0302@gmail.com

**Keywords:** hip fracture, discharge destination, gait speed, balance

## Abstract

*Background and Objectives*: The majority of patients who undergo hip fracture surgery do not recover their former level of physical function; hence, it is essential to establish a specific rehabilitation strategy for maximal functional recovery of patients after a hip fracture. Knowing which indicators of physical function in hip fracture patients have a significant impact on the decision regarding the place or timing of discharge would make it possible to plan and prepare for discharge as soon as possible. Therefore, this study aimed to investigate the relationship between physical function and discharge destination for older adult patients with hip fracture. *Materials and Methods*: In this retrospective cohort study, 150 hip fracture patients (mean age 78.9 ± 10.6 years) between January 2019 and June 2021 were enrolled. Patients were categorized into two groups according to their discharge destination, either home or facility. Demographic and disease-related characteristic data were collected from the medical records. All the patients completed performance-based physical function tests including the 10 Meter Walk Test (10MWT), Timed Up and Go test (TUG), Koval’s grade, and Berg Balance Scale (BBS) at the start of rehabilitation and at discharge. A backward stepwise binary logistic regression analysis was then performed to determine the independent factors of the discharge destination. *Results*: The home discharge group had a significantly lower Koval’s grade, lower TUG, higher BBS both at baseline and discharge, and younger age. Backward stepwise logistic binary regression analysis showed that TUG, BBS, and 10MWT at baseline and discharge were significant variables affecting the discharge destination after hip fracture. *Conclusions*: These results demonstrate that balance and gait in older adult patients with hip fractures are highly influential factors in the determining the discharge destination.

## 1. Introduction

As life expectancy has risen worldwide, hip fractures have become one of the most important health problems among older adults in developed countries. The annual incidence of hip fractures is increasing each year, and the incidence is estimated to rise globally from 1.7 million cases in 1990 to 6.3 million cases by 2050 [1]. According to a previous study in the U.S., 50% of hip fracture patients did not regain their pre-fracture levels of mobility and independence, and 25% needed long-term care at home or in a facility. Furthermore, a hip fracture causes 30% of patients to pass away a year later or to suffer a major functional impairment or disability [2].

Most hip fracture patients require help getting out of bed, standing, and walking after surgery, which negatively affects their quality of life and reduces their functional levels [3]. Therefore, hip fractures often require inpatient rehabilitation, and the goal of rehabilitation in patients with hip fractures is to restore function to pre-injury levels. Functional recovery following hip fracture surgery is a major concern for both providers and patients and is considered a major factor in preparing the timing of discharge and its destination.

According to a study by Wang et al. [4], patients with hip fractures who have a Functional Independence Measure (FIM) motor score of 58 at discharge can be classified as having a community discharge destination. FIM motor was significantly more effective than FIM cognition and equally effective as FIM total in differentiating patients who were discharged to the community from those who were discharged to a facility following inpatient rehabilitation for a hip fracture.

Another study demonstrated that a FIM cognitive score of 24 on admission could be used to classify community discharge destinations in hip fracture patients who live alone [5]. Indeed, for patients with hip fractures and cognitive impairments, including delirium or dementia, it is often difficult to assess the efficacy and treatment compliance with rehabilitation. A previous study had excluded patients with moderate to severe cognitive impairments while examining functional recovery and discharge destination after hip fractures [6]. However, the study did not include rehabilitation treatment and only investigated the relationship between functional outcomes and discharge destination.

To our knowledge, there is no study examining whether the actual performance-based physical function influences the discharge destination after rehabilitation in patients without cognitive impairment. Therefore, this study aimed to investigate and identify the relationship between physical performance measures and discharge destination for patients with hip fractures.

## 2. Materials and Methods

### 2.1. Study Design and Participants

This retrospective study was conducted on patients with hip fractures who underwent surgery and were transferred to the inpatient rehabilitation ward of the Department of Rehabilitation Medicine at OO between January 2019 and June 2021. Data including age, gender, type of medical insurance, operation type, Charlson Comorbidity Index (CCI), time from surgery to Rehabilitation Medicine (RM) transfer, and RM hospitalization period, were collected from the medical records. The medical insurance of South Korea is a two-tiered system. Approximately 96% of people are covered by the wage-based, contributory health insurance program, whereas the lowest-income population is covered by a medical aid program [7]. The results of performance-based physical function tests, including the 10 Meter Walk Test (10MWT), Timed Up and Go test (TUG), and Berg Balance Scale (BBS), were also collected. All the patients used rolling walkers during the 10MWT and TUG due to the risk of falling, and functional outcomes were assessed by the same skilled physical therapist at the start of rehabilitation and at discharge. Patients with severe cognitive impairment (unable to obey commands beyond one step) who were unable to perform the performance-based physical function tests were excluded. As a result, 56 out of 206 eligible patients for this study were excluded. Patients were categorized into two groups according to their discharge destination, namely the home environment (home group, *n* = 99) and the long-term care facility (facility group, *n* = 51). Demographic and disease-related characteristic data and performance-based physical function were compared between the two groups.

From the second day after surgery, all patients underwent early rehabilitation treatment, which consisted of ice packs, transcutaneous electrical nerve stimulation, bed mobility, a passive range of motion exercises, and starting to stand with a tilt table within 3 or 5 days. The patients then practiced walking or standing using parallel bars under the supervision of a physical therapist. Depending on the surgical method and the patient’s condition, there were variations in when the patient could bear weight, but no patient had limited weight bearing.

After completing these early rehabilitation sessions, the patients were transferred to the Department of Rehabilitation Medicine and received intensive rehabilitation treatment, including gait training with an anti-gravity treadmill, progressive resistance strengthening exercises, balance training, and stair climbing. The first part of intensive rehabilitation treatment involved progressive gait training exercise using a lower body positive pressure treadmill (Alter G anti-gravity treadmill, Model M320; Alter G Inc., Fremont, CA, USA). Patients began walking at a comfortable speed with 50% body weight support (BWS) and progressed by adjusting the gait speed and/or the degree of BWS. The second part involved progressive resistance strengthening exercises of the lower extremities, including leg extension, and curl and hip abduction, starting from 10 to 20% of each patient’s one-repetition maximum, using air-resistance equipment (HUR Co, Kokkola, Finland). Each session consisted of 3 sets of 15 repetitions. The third part involved a one-on-one rehabilitation treatment between patients and physical therapists, including sit-to-stand/lateral step-up exercises, stepping exercise, task-oriented balance training, and stair climbing. The intensity of exercise for each patient gradually increased until they could perform adequately. This rehabilitation program was administered for approximately 1.5 h/day, 5 days per week, and was supervised by physical therapists. After intensive rehabilitation therapy, the patient was evaluated by a multidisciplinary team, and depending on the patient’s condition, the hospitalization period may be extended, or the patient may be discharged or transferred.

The study protocol was approved by the Institutional Review Board at our hospital (OO 2019-12-018).

### 2.2. Demographic and Disease-Related Characteristics Data

Charlson Comorbidity Index: The Charlson Comorbidity Index (CCI) is a method of classifying patient comorbidities based on the International Classification of Diseases (ICD) diagnosis codes. The CCI was designed to predict one-year mortality based on a weighted composite score, where comorbidities are weighted from 1 to 6 for mortality risk and disease severity, and then summed to form the total CCI score. The total score ranges from 0 to 37 points. The higher the score, the more likely the predicted outcome is to result in mortality [8].

### 2.3. Performance-Based Physical Function

Koval grade: The Koval grade is used to assess walking dependency, in which individuals are classified into seven grades as follows: independent community ambulators (Grade 1), community ambulators with a cane (Grade 2), community ambulators with walkers/crutches (Grade 3), independent household ambulators (Grade 4), household ambulators with a cane (Grade 5), household ambulators with walkers/crutches (Grade 6), and nonfunctional ambulators (Grade 7) [9].

Berg Balance Scale: The Berg Balance Scale (BBS) was developed as a performance-oriented measure of balance in older adults [10]. The BBS is a testing tool that assesses an individual’s capacity for safe balance by having them carry out functional tasks such as reaching, bending, transferring, and standing, and it incorporates most components of postural control. The BBS consists of 14 items scored on an ordinal scale ranging from 0 to 4 (0 indicating the lowest level of function), with a maximum total score of 56. Higher scores indicate better balance and functional independence with respect to the activities tested.

The 10 Meter Walk Test: The 10 Meter Walk Test (10MWT) is a simple assessment to measure locomotor capacity and a performance measure used to evaluate walking speed in millimeters per second over a short distance. It measures how long it takes a person to walk a distance of 10 m at their usual pace, giving important details on their functional mobility, gait, balance, and endurance. In this test, participants walk 10 m, and the time taken to walk the middle 6 m is measured, to exclude acceleration and deceleration [11].

Timed Up-and-Go test: The Timed Up-and-Go test (TUG) starts from a seated position in a standard armchair (seat height = 46 cm). The patient is then instructed to get up from the chair, walk 3 m (with their usual walking aid) to a line of colored tape placed on the floor, turn around, return to the chair, and sit back down. The stopwatch starts when the patient’s buttocks lift from the seat and stops when the buttocks retouch the seat [12].

### 2.4. Statistical Analysis

Analyses were performed using the Statistical Package for the Social Sciences version 22.0 (IBM, Armonk, NY, USA) and MedCalc Version 22.0 (MedCalc software Ltd., Ostend, Belgium). The differences in baseline demographics and disease-related characteristics are presented as means and 95% confidence intervals (CI), or percentages. The differences in performance-based physical function between group of patients who were discharged to a facility compared to those discharged home were analyzed using independent sample t-tests at each time point (beginning of rehabilitation and discharge after rehabilitation). Changes in the measured variables, such as Koval’s grade, BBS, 10MWT, and TUG, are presented as the mean ± standard deviation (SD). Moreover, backward stepwise binary logistic regression was performed using the values derived from significant results to investigate the independent factors in patients who were discharged home. In addition, we drew a Receiver Operation Characteristic (ROC) curve and estimated an Area Under the ROC Curve (AUROC) using the sensitivity and specificity of each performance-based physical function test. The cut-off values of continuous variables based on the results of each test were calculated by the ROC curve. Optimal cut-off values were determined using the Youden index, which is the difference between the true positive rate (sensitivity) and the false positive rate (1-specificity) in the ROC curve. All *p* values were two-sided, and *p* < 0.05 was considered statistically significant.

## 3. Results

The patient demographics and disease-related characteristics are presented in Table 1. This study included 150 hip fracture patients with an average age of 78.9 ± 10.6 years. In total, 99 patients were classified into the home discharge group (66%), and 51 were classified into the long-term care facility group (34%). The average age of the home group was significantly lower than that of the facility group, as was the CCI score, and the mean duration of RM hospitalization for rehabilitation. No differences were found in other characteristics between the groups.

**Figure 1:**
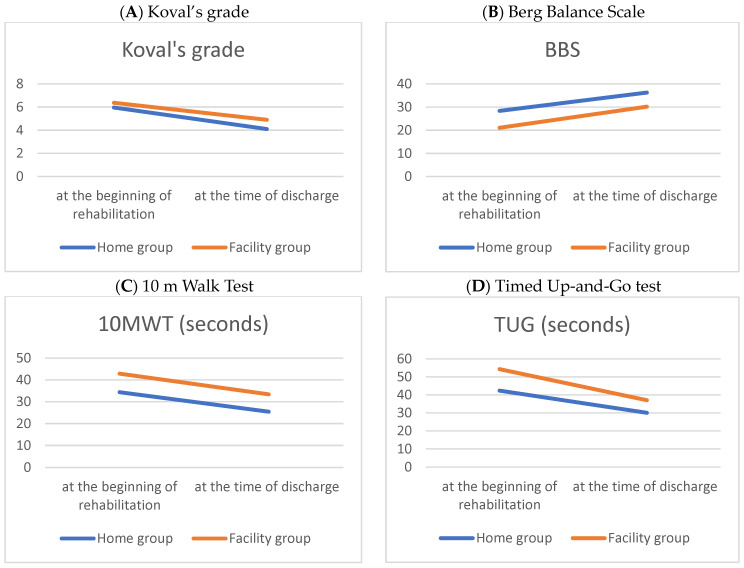
Line graphs of parametric performance-based physical functions during hospitalization period in patients with a hip fracture.

Table 2 and Figure 1 compare the performance-based physical function outcomes between the home and facility groups at the beginning and at discharge, following the hip fracture surgery. Following intensive rehabilitation treatment, improvements in physical function were verified in both groups at discharge. The home group showed better performance-based physical function at the beginning and discharge than the facility group.

In the performance-based physical function evaluation, the Timed Up-and-Go test showed a significant change, and it is presumed that the facility group showed a greater amount of change because the initial evaluation score was lower compared to the home group (Table 3).

Table 4 presents the independent factors for patients discharged home. The results of backward stepwise logistic regression analysis indicate that pre-Koval’s grade, 10MWT, BBS, and TUG are significant independent factors in the group of patients discharged home.

**Figure 2:**
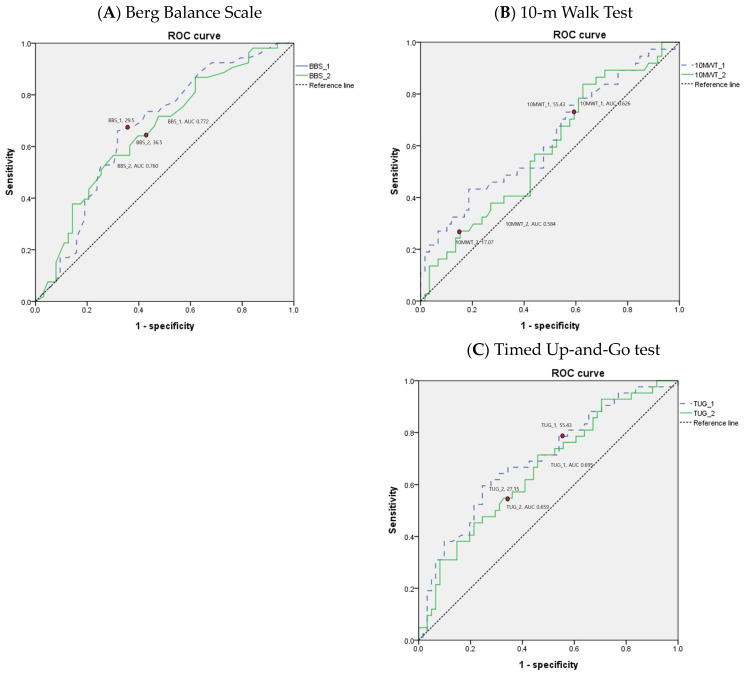
Receiver operating characteristic (ROC) curves for performance-based physical function tests in older adult patients with hip fracture.

The values of the AUC for performance-based physical function tests are shown in Table 5. Areas under the ROC curves for BBS, 10MWT, and TUG suggest that the performance-based physical function tests yield the best overall differentiation of the patients discharged to the home versus those discharged to the long-term care facility following inpatient rehabilitation for hip fracture. The ROC curves of the performance-based physical function tests in older adult patients with hip fracture are depicted in Figure 2.

## 4. Discussion

Overall, the results of this study demonstrate that balance and gait physical function are the most influential factors affecting the decision regarding discharge destination for patients with hip fractures. Among several gait parameters, a previous study suggested that gait speed may be the most useful in assessing gait function. Normal gait speed is a task developed through physiotherapy and exercise, requiring multiple abilities such as endurance, muscle strength, balance, coordination, and weight-bearing control [13]. Kline et al. [14] found that gait speed after a hip fracture was affected by the strength of the operated hip extensor and abductor muscles. In fact, a decrease in hip abduction the moment after a hip fracture can lead to abnormal hip motion, resulting in a slow gait. Similarly, Kubota et al. [15] demonstrated the importance of improving hip abduction strength after surgery to allow patients with a hip fracture to walk with a higher maximal hip abduction moment in the stance phase.

Gait speed is generally determined by stride length and stride time [16]. A person with weak hip extensors will take smaller steps to reduce the amount of hip flexion necessary for initial contact, resulting in slower overall gait to allow the limbs time to stabilize. Furthermore, the hip abductor muscles contribute to various actions, including pelvic stabilization during running and walking. During the single-limb support phase of walking, the hip is stabilized in the coronal plane by the hip abductor muscles. When the hip abductor muscles are weak, the pelvis becomes unsteady, making it difficult to walk or stand on one leg [17,18]. Hence, the patient’s gait ability is an important basis for determining the discharge destination of patients with hip fractures. Taking this finding into consideration, lower extremity strengthening through hip abductor and hip extensor training has important clinical implications, as it can increase functional mobility in patients with hip fractures by improving gait speed. Also, these findings highlight the importance of a targeted exercise program focused on strengthening the hip abductors and hip extensors for home discharge after hip fracture surgery.

The importance of balance and patients’ walking capacity and efficiency in the rehabilitation of older adult patients with hip fractures was further supported by the TUG test findings. The clinical significance of the TUG test lies in its ability to improve several important mobility skills, including straight-ahead gait, turning, and sit-to-stand transitions, all of which require balance control [19] not only for maintaining postural stability, but also for ensuring safe performance of daily mobility-related activities such as walking, rising from a chair, and turning. Physical function was found to be considerably enhanced when balance exercises were added within a year of hip fracture surgery in a meta-analysis encompassing eight RCTs and 752 individuals. In a different meta-analysis encompassing nine RCTs and 872 hip fracture patients, the rehabilitation plus balancing exercise group’s total function improved more [20,21].

In general, it is assumed that a decline in balance causes the falls that precede a hip fracture [22]. The reduced ability to balance is reported to be age-affected, as maintaining postural control requires the coordination of multiple body systems, including the visual, somatosensory, vestibular, and motor systems, and peripheral proprioceptive information [23]. Additionally, balance is one of the skills most affected by abnormal proprioception, and its deficit may reduce the quality of life and increase the risk of falling [24,25,26]. Moreover, since proprioceptive information is essential for movement programming, abnormal proprioceptive signals impact both motor control and sensory function [27]. These findings are consistent with the results of this study, which found that people with poor balance have a high risk of falling again. So, when choosing a discharge destination, they should prioritize alternative rehabilitation facilities before their own house. Therefore, programs focused on regaining balance and proprioception should be planned to continue regularly for patients whose ability to balance has not significantly improved at the time of discharge.

Additionally, the BBS, 10MWT, and TUG demonstrate good suggestive tools for differentiating patient discharge settings following inpatient rehabilitation for older adult patients with hip fractures. At the beginning of rehabilitation, a BBS score of >26, a 10MWT time of ≤53.09 s, and a TUG time of ≤55.91 s may each represent thresholds for an increased likelihood of home discharge. If the cutoff value is not met, there is a high possibility of being discharged to a long-term care facility, so rehabilitation therapy should focus on improving gait speed and balance. At the time of discharge after rehabilitation treatment, a BBS score of >31, a 10MWT time of ≤17.03 s, and a TUG time of ≤27.1 s may each represent thresholds for an increased likelihood of home discharge.

Planning discharge destinations for older adult patients with hip fractures can be challenging. Factors beyond the patient’s individual insurance status, such as social support, economic status, geographic area of residence, and hospital characteristics, play a role in influencing the discharge destination after rehabilitation treatment. Therefore, as various factors are related to the discharge destination, our hospital employs a multidisciplinary team approach. At our hospital, multidisciplinary team evaluations, which include physical functional scores based on performance evaluations related to activities of daily living, are conducted by rehabilitation medicine doctors, nurses, therapists, and social workers in order to plan and recommend discharge destinations for patients. Riemen AH et al. [28] found that hip fracture patient-centered multidisciplinary care provides the best outcomes beyond just patient care. This has positive implications for patients, reduces readmission rate and length of hospital stay, and improves patient satisfaction. Therefore, it is now recognized that good multidisciplinary teamwork and care are essential for these patients.

Lastly, up to 40% of people with a hip fracture experience some form of cognitive impairment, which may include delirium, dementia, or other post-operative cognitive deterioration [29]. Among these, patients with severe cognitive impairment are at a greater risk of comorbidities such as surgical site infections, urinary tract infections, pressure sores, and respiratory infections, and they are more likely to require long-term care [30]. Therefore, the discharge destination of these hip fracture patients may be determined solely by severe cognitive impairment. The purpose of this study was to identify the factors that have the greatest impact on the discharge destination of hip fracture patients through performance-based physical function assessments. As such, patients with severe cognitive impairment (unable to obey commands beyond one step) who had difficulty performing functional assessments were excluded.

Our study has limitations which should be taken into consideration. First, patients with severe cognitive impairment were excluded, meaning we could not confirm the discharge destination according to the degree of cognitive impairment. Furthermore, a follow-up prospective study is required to establish the discharge destination based on the degree of cognitive impairment, as there was no objective measurement of the patient’s basic cognitive score. Second, the sample was taken from one hospital in OO. We believe that our institution can represent this region because it is the only hospital in OO that functions as a tertiary general hospital, but it may not be possible to generalize to other developed and emerging cohorts and diverse health service delivery systems. Third, we did not incorporate other clinically relevant factors that may influence discharge decisions, such as social support, economic status, or home environment. Fourth, although we did not find significant differences in the hip fracture type among the patient groups, our study did not focus on fracture type. Different hip fracture types could potentially have a significant impact on patients’ physical function and discharge destinations. Thus, future research should be planned in consideration of these factors to provide a more comprehensive understanding of the multifaceted nature of discharge planning in patients with hip fractures. Fifth, the small sample size may have limited our ability to investigate the interactions between outcomes and predictors. A large-scale cohort study is needed to evaluate the clinical effectiveness of using the cut-off values of BBS, 10MWT, and TUG as a practical tool to guide rehabilitation plans and/or as additional information in discharge planning decisions for patients with hip fracture. Also, further research is required to establish the optimum type and intensity of exercise for enhanced mobility and balance after a hip fracture.

## 5. Conclusions

This study demonstrated that it is necessary to confirm and monitor gait speed and balance before discharge in order to determine the discharge destination of patients with hip fractures after intensive rehabilitation treatment. It has clinical implications in that it presents a cut-off value for an objective method to identify the patient’s gait speed and balance abilities. Rehabilitation following hip fracture surgery should concentrate on enhancing balance and strengthening the lower extremities with hip abductor and hip extensor exercises. Furthermore, organizing the discharge of older adult patients with hip fractures could be challenging due to various factors influencing both discharge destination and the planning process. It is crucial to work in conjunction with a multidisciplinary team to ensure a well-coordinated discharge plan. The information obtained from this study will be useful for determining discharge plans and setting rehabilitation goals.

## Figures and Tables

**Table 1:** Demographic and disease-related characteristics of the participants (N = 150).

Variables	Home Group (*n* = 99)	Facility Group (*n* = 51)	*p*-Value
Age (years)	77.4 ± 11.6	81.1 ± 7.7	0.024 *
Sex, males/females	23 (23.2)/76 (76.8)	8 (15.7)/43 (84.3)	0.666
BMI (Kg/m^2^)	22.4 ± 3.5	21.8 ± 4.1	0.357
Type of insurance			0.205
Medical aid program	11 (11.1)	10 (19.6)	
Health insurance program	88 (88.9)	41 (80.4)	
Fracture side			0.478
Right	48 (48.5)	22 (43.1)	
Left	51 (51.5)	29 (56.9)	
Fracture type			0.234
Femur neck	40 (40.4)	23 (45.1)	
Intertrochateric	40 (40.4)	24 (47.1)	
Subtrochanteric	19 (19.2)	9 (17.6)	
Operation type			0.465
Bipolar hemiarthroplasty	13 (13.1)	11 (21.6)	
Total hip replacement arthroplasty	20 (20.2)	11 (21.6)	
Reduction and internal fixation	64 (64.6)	29 (56.9)	
Charlson Comorbidity Index	2.43 ± 1.32	3.02 ± 1.41	0.015 *
Time from surgery to RM transfer (days)	21.1 ± 5.7	21.8 ± 6.2	0.511
Hospitalization period at RM (days)	11.7 ± 2.9	14.0 ± 6.1	0.003 **

Values represent mean ± standard deviation or number (%) of cases. Abbreviations: BMI, Body Mass Index; RM, rehabilitation medicine; * *p* < 0.05, ** *p* < 0.01.

**Table 2:** Performance-based physical function at beginning and at discharge after hip fracture surgery.

Variables	Home Group (*n* = 99)	Facility Group (*n* = 51)	*p*-Value
Pre-Koval’s grade	1.77 ± 1.48	2.57 ± 1.80	0.009 **
Koval’s grade ^1^	5.96 ± 0.81	6.37 ± 0.70	0.003 **
BBS ^1^	28.36 ± 13.42	21.06 ± 13.55	0.003 **
10MWT (S) ^1^	34.37 ± 20.19	42.87 ± 25.99	0.005 **
TUG (S) ^1^	42.38 ± 28.69	54.31 ± 28.61	0.004 **
Koval’s grade ^2^	4.10 ± 1.87	4.90 ± 1.51	0.001 **
BBS ^2^	36.27 ± 12.21	30.18 ± 11.58	0.005 **
10MWT (S) ^2^	25.45 ± 15.60	33.40 ± 14.94	0.004 **
TUG (S) ^2^	30.07 ± 14.90	37.06 ± 14.91	0.009 **

Values represent mean ± standard deviation; BBS, Berg Balance Scale; 10MWT, 10 Meter Walk Test; TUG, Timed Up-and-Go test; ^1^ at the beginning of rehabilitation; ^2^ at the time of discharge after rehabilitation; ** *p* < 0.01.

**Table 3:** Changes in performance-based physical function.

Variables	Home Group (*n* = 99)	Facility Group (*n* = 51)	*p*-Value
ΔKoval’s grade	−1.78 ± 1.77	−1.30 ± 1.39	0.163
ΔBBS	7.91 ± 7.04	9.12 ± 8.10	0.362
Δ10MWT (S)	−10.50 ± 13.56	−11.15 ± 25.12	0.882
ΔTUG (S)	−13.28 ± 24.50	−20.37 ± 27.35	0.040 *

Values represent mean ± standard deviation; BBS, Berg Balance Scale; 10MWT, 10 Meter Walk Test; TUG, Timed Up-and-Go test; * *p* < 0.05.

**Table 4:** Results of backward stepwise binary logistic regression analysis to investigate independent factors in patients discharged home (adjusted for age and Charlson Comorbidity Index).

Variables	B	S.E.	Wald	*p*	Odds Ratio	95% CI
preKoval’s grade	0.196	0.082	5.684	0.017 *	1.309	1.035–1.428
BBS (S) ^1^	−0.036	0.011	10.925	0.001 **	0.965	0.944–0.985
10MWT (S) ^1^	0.026	0.009	8.905	0.003 **	1.026	1.009–1.043
TUG (S) ^1^	0.016	0.005	8.278	0.004 **	1.035	1.008–1.058
BBS (S) ^2^	−0.039	0.014	7.345	0.007 **	0.962	0.935–0.989
10MWT (S) ^2^	0.035	0.014	6.205	0.013 *	1.036	1.007–1.064
TUG (S) ^2^	0.044	0.013	10.883	0.001 **	1.045	1.018–1.073

Abbreviations: BBS, Berg Balance Scale; TUG, Timed Up-and-Go test; 10MWT, 10 Meter Walk Test; ^1^ at the beginning of rehabilitation; ^2^ at the time of discharge after rehabilitation; * *p* < 0.05 ** *p* < 0.01.

**Table 5:** Optimal cutoff, sensitivity, specificity, and area under the ROC curve of performance-based physical function tests for predicting discharge to home in older adult patients with hip fracture.

Variables	Cutoff	Sensitivity (%)	Specificity (%)	AUC	PPV	NPV	*p*-Value
BBS ^1^	≤26	64.5	60.3	0.626	54.5	69.7	0.001 **
BBS ^2^	≤31	56.6	69.8	0.660	61.2	65.7	0.002 **
10MWT (S) ^1^	>53.09	33.3	87.9	0.632	62.9	68.1	0.003 **
10MWT (S) ^2^	>17.03	88.2	36.1	0.648	53.6	78.6	0.005 **
TUG (S) ^1^	>55.91	55.6	76.8	0.670	60.6	72.9	<0.001 **
TUG (S) ^2^	>27.1	76.0	54.1	0.699	57.6	73.3	<0.001 **

Abbreviations: BBS, Berg Balance Scale; 10MWT, 10 Meter Walk Test; TUG, Timed Up-and-Go test; AUC: area under curve; ROC: receiver operating characteristic curve; PPV, Positive Predictive Value; NPV, negative predicative value; ^1^ at the beginning of rehabilitation; ^2^ at the time of discharge after rehabilitation treatment; ** *p* < 0.01.

## Data Availability

Data are available upon request.
